# Chitosan-Carboxymethyl Tamarind Kernel Powder Interpolymer Complexation: Investigations for Colon Drug Delivery

**DOI:** 10.3797/scipharm.0908-10

**Published:** 2009-12-03

**Authors:** Gurpreet Kaur, Subheet Jain, Ashok K. Tiwary

**Affiliations:** Department of Pharmaceutical Sciences and Drug Research, Punjabi University, Patiala, 147002, India

**Keywords:** Colon release, Microbially triggered system, Interpolymer complexation, Budesonide, Polysaccharide based colon delivery

## Abstract

The present study was aimed at evaluating the possible use of inter polymer complexed (IPC) films of chitosan (CH) and carboxymethyl tamarind kernel powder (CMTKP) for colon release of budesonide. Viscosity analysis of the supernatant liquid obtained after reacting CH and CMTKP in different proportions revealed 40:60 to be the optimum stoichiometric ratio. The FTIR spectra of IPC films formed from 50:50 or 40:60 ratio of CH:CMTKP did not reveal any reduction in the peaks at 1560cm^−1^ and 1407cm^−1^ after exposure to pH 1.2, suggesting resistance of the interaction between −COO^−^ groups of CMTKP and −NH_3_^+^ groups of CH to gastric pH. Tablets containing Avicel pH 102 as diluent and coated to a weight gain of 10%, w/w with aqueous solutions of 40:60 or 50:50 ratio of CH:CMTKP did not release budesonide in pH 1.2 buffer. Histopathology of the rat colon after oral administration of these IPC film coated tablets revealed significantly greater (p<0.05) reduction in TNBS-induced ulcerative colitis as compared to that after administration of uncoated tablets. The Cmax of budesonide achieved after oral administration of these IPC film coated tablets was comparable to that observed after administration of uncoated tablets. The results strongly indicate versatility of CH-CMTKP IPC films to deliver budesonide in the colon.

## Introduction

Colon delivery for achieving either maximum drug absorption or local action is being extensively investigated over the past two decades. The main advantages associated with colon delivery is that the colon offers a near neutral pH, reduced digestive enzyme activity, a long transit time and increased responsiveness to absorption enhancers. However, due to its location at the distal part of the alimentary tract, the colon is particularly difficult to access. In addition, wide range of pH values and different enzymes present throughout the GIT, through which the dosage form has to travel before reaching the target site, further complicates the reliability and delivery efficiency. The classical approaches make use of polymers with pH-dependent [1] or pH-independent release characteristics [2]. Drug release in the former case is easily influenced by nature of diet. Further, physiologically, a highly alkaline pH of 7.4 of the small intestine often contributes to premature drug release and failure of the pH-dependent release systems before reaching the colon [3]. The pH-independent release systems suffer from the drawback of incomplete drug release and have to be combined with other polymers that are either soluble at colonic pH or are capable of being degraded by colonic bacteria.

The recent innovations make use of biodegradable polymer combinations that are cross linked with each other or with ions in order to render them insoluble in acidic pH. Cross linking of hydroxyl groups of guar gum with ester groups of trisodium triphosphate is reported to yield di-polymer phosphate ester type of interaction, which reduces the swelling of guar gum [4]. Carboxymethyl konjac glucomannan-chitosan polyelectrolyte beads have been reported for the delivery of protein delivery owing to its pH sensitive drug release [5].

CH is a linear polysaccharide obtained from deacetylation of chitin. Due to amino groups, it carries a net positive charge and can be easily cross-linked with other anions, oppositely charged drugs and polymers [6, 7] Beads prepared by cross linking CH with sodium alginate have been observed to protect drug release in stomach thus, serving as potential drug carriers for small intestine or colon [8].

Tamarind kernel powder (TKP) is derived from the seeds of *Tamarindus indica* Linn. commonly found in India and South East Asia. TKP is one of the cheapest gums available in India. However, because of several drawbacks, such as unpleasant odor, dull color, presence of water insolubles, low solubility in cold water, and fast biodegradability, its various derivatives e.g. acetyl, hydroxyalkyl and carboxymethyl [9, 10] have been prepared. Carboxymethyl tamarind kernel powder (CMTKP) is a carboxymethylated derivative of tamarind kernel powder. The incorporation of a carboxylic group imparts anionic character to CMTKP, thus allowing the formation of interpolymer complex between CMTKP and cationic polymers.

Budesonide, a second generation glucocorticoid is used in the treatment of IBD. It has the highest affinity for glucocorticoid receptor as compared to other steroids (hydrocortisone, prednisolone, dexamethasone) and it does not reduce the cortisol levels. The pharmacokinetic profile of budesonide favors a high topical efficacy because of rapid uptake by mucosal tissue and enhanced receptor binding properties [11]. In the light of these facts, a dosage form capable of delivering budesonide in a sustained manner during its transit through the lower part of GIT can be envisaged to provide a new paradigm in treating IBD more effectively.

In the light of above facts, it was proposed to formulate colon release tablets of budesonide by coating them with aqueous mixtures containing CH and CMTKP in order to yield interpolymer complexed (IPC) film coated tablets. The characterization of the IPC films was done by FTIR studies. The effect of varying the composition of CH and CMTKP in the coating solution on the *in vitro* release of budesonide from tablets was investigated. Further, the formulated budesonide tablets were evaluated for their pharmacokinetic and pharmacodynamic performance after oral administration to rats.

## Results and Discussion

### Preparation and Characterization of CMTKP

**Fig. 1.**
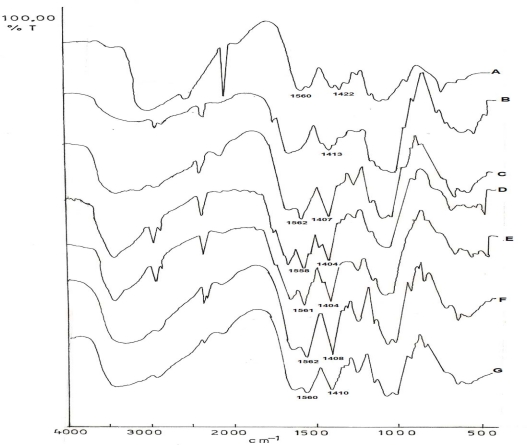
FTIR spectra of: CH powder (A), CMTKP powder (B); films prepared by interacting CH and CMTKP in the ratio of 70:30 (C), 60:40 (D), 50:50 (E), 40:60 (F) or 30:70 (G)

The prepared CMTKP was characterized by Fourier Transform Infra Red (FTIR) spectroscopy. The appearance of a peak at 1413 cm^−1^ indicated the presence of −COO^−^ ions (representing C=O stretch of −COO^−^) in the CMTKP specimen (Fig.1B). This peak was absent in TKP sample thus, indicating that carboxymethylation had been successfully carried out. The degree of substitution was calculated according to the method described by Whistler, 1963 [12] and was found to be 0.2.

### Studies on CH-CMTKP complex

#### Stoichiometry of the CH-CMTKP complex

**Fig. 2.**
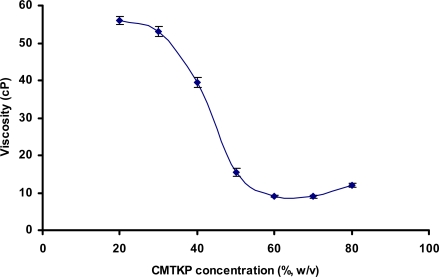
Viscosity of the supernatant obtained after centrifugation of admixed aqueous solutions containing different ratios of CH and CMTKP

Fig. 2 shows the viscosity of the supernatants obtained after mixing solutions of CH with CMTKP solutions in different ratios. CMTKP bears a negative charge owing to the presence of −COO^−^ group introduced in CMTKP by carboxymethylation. CH bears a net positive charge due to presence of −NH_3_^+^ groups. As a result, both polymers undergo spontaneous reaction when mixed together, resulting in formation of a solid mass. The viscosity of the supernatant obtained after mixing aqueous solutions of CH and CMTKP was observed to decrease as the proportion of CH in CH-CMTKP mixtures decreased. Moustafine *et al.* (2006) reported the viscosity to drop to minimum when the molar ratio between Eudragit E PO and Eudragit L 100-55 was unity [13]. The minimum viscosity of the supernatant obtained by reacting CH and CMTKP in the ratio of 40:60 indicated maximum interaction.

### Characterization of CH-CMTKP IPC films

#### Spectroscopic attributes

Fig. 1 depicts the FTIR spectrographs of CH, CMTKP and IPC films formed by interacting various ratios of CH and CMTKP. It is evident from Fig. 1A that CH powder (85% deacetylation) exhibited a peak at 1560 cm^−1^ indicating presence of −NH_3_^+^ ions [14]. The peak at 1422 cm^−1^ (Fig. 1A), suggesting presence of −COO^−^ ions could have arisen due to 15% acetylation of CH powder. The FTIR spectrum of CMTKP (Fig. 1B) exhibited peaks at 1413 cm^−1^ suggesting the presence of −COO^−^ groups that were introduced by carboxymethylation of TKP. The IPC films formed by interacting different ratios of CH:CMTKP showed a peak at 1407 cm^−1^ indicating the presence of −COO^−^ ions (representing C=O stretch of −COO^−^). Further, the presence of a peak at 1560 cm^−1^ could be ascribed to the existence of −NH_3_^+^ (representing N-H stretch of −NH_3_^+^) in CH molecule (Figs. 1C–G). Thus, the presence of −COO^−^ groups and −NH_3_^+^ groups in the IPC films could be ascribed to the existence of NH_3_^+^COO^−^ complex between CH and CMTKP molecules thus, leading to interpolymer complexation.

**Fig. 3.**
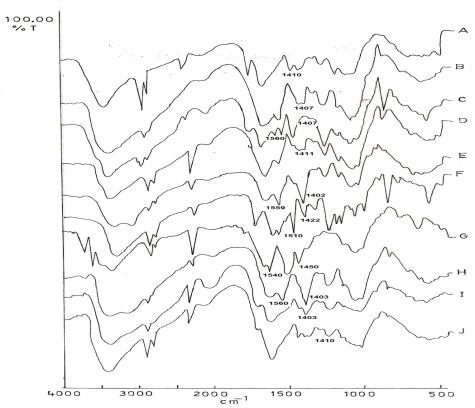
FTIR spectra of IPC films containing different CH:CMTKP ratios after exposure to different pH media: 70:30 (pH 1.2) (A); 70:30 (pH 7.4) (B); 60:40 (pH 1.2) (C); 60:40 (pH 7.4) (D); 50:50 (pH 1.2) (E); 50:50 (pH 7.4) (F); 40:60 (pH 1.2) (G); 40:60 (pH 7.4) (H); 30:70 (pH 1.2) (I) or 30:70 (pH 7.4) (J).

All these IPC films were exposed to pH 1.2 for 2 h followed by buffer pH 7.4 for 22 h. The peak at 1407 cm^−1^ corresponding to −COO^−^ was observed to considerably decrease in intensity and the peak at 1560 cm^−1^ corresponding to −NH_3_^+^ ions was found to be completely abolished in the IPC films comprising of 70:30 ratio of CH:CMTKP after exposure to pH 1.2. This suggested total breakdown of COO^−^ NH_3_^+^ linkages in these IPC films (Fig. 3A). The spectrographs were observed to be similar in nature after exposure of these films to buffer pH 7.4 (Fig. 3B). The spectrographs of IPC films containing 60:40 ratio of CH:CMTKP showed reduced intensity of peaks at 1560 cm^−1^ and 1407 cm^−1^, thus indicating weakening of NH_3_^+^COO^−^ interaction after exposure to acidic pH (Fig. 3C). Further, the −NH_3_^+^ ion peak at 1560 cm^−1^ disappeared completely after treatment with pH 7.4 buffer for 22 h (Fig. 3D). The spectrograph of IPC film prepared by using 30:70 ratio of CH:CMTKP revealed absence of peak at 1560 cm^−1^ after treatment with pH 1.2 and 7.4, thus indicating complete breakdown of COO^−^NH_3_^+^ bonds (Fig. 3I,J). However, the IR spectrographs of films containing 50:50 (Figs. 3E and F) or 40:60 CH:CMTKP (Fig. 3G and H) did not reveal any change in the intensity of peaks at 1560 cm^−1^ and 1407 cm^−1^ after treatment with buffer 1.2 and 7.4. This indicated that carboxylate linkages in IPC films prepared from 50:50 or 40:60 were resistant to pH 1.2 and 7.4.

#### Swelling Index determination

The CH-CMTKP IPC films were found to exhibit swelling in acidic medium as well as in basic medium (Tab. 1). It is known that a decrease in charge density of the cross-linker decreases the cross-linking density, which leads to swelling. This swelling is favored by the protonation and repulsion of chitosan free ammonium groups. Highly acidic pH of the stomach dissociates the ionic linkages followed by dissolution of the network. This in turn is reported to result in fast drug release [15]. However, the IPCs exhibited considerably low swelling in pH 7.4. The swelling index was found to decrease to a limiting value when the CMTKP concentration was increased in the CH-CMTKP complex to 60%, w/w. However, further increase in CMTKP concentration increased the swelling index. The introduction of carboxymethyl groups in TKP has been reported to increase the swelling of TKP [16]. Thus, the IPC films comprising 30:70 ratio of CH-CMTKP showed increased swelling as compared to those containing 40:60 ratio of CH-CMTKP.

### Physical Evaluation of Tablets

The average weight of uncoated core tablets was 24.67 ± 1.10 mg. The acceptance value calculated was 11.98% which was well below the maximum 15% USP tolerance limit [17]. Hence, the tablets passed the weight variation test. Hardness of the tablets was 4.5 ± 0.5 kg/cm^2^ and friability was found to range from 0.36 to 0.46%, w/w. The axial and radial diameters, respectively, ranged from 1.75 to 1.80 mm and 3.98 to 4.02 mm. The uncoated tablets prepared by using 10%, w/w Eudragit L100-55 or CH:CMTKP as a binder started showing signs of cracking within 30 min of exposure to 0.1M HCl.

The average weight of coated tablets was 27.20 ± 1.25 mg. The acceptance value calculated was 11.31%. Hence, the tablets passed the weight variation test. The axial and radial diameters of coated tablets, respectively, ranged from 2.02 to 2.08 mm and 4.01 to 4.15 mm. Although, these tablets exhibited swelling, they didn’t soften or crack after exposure to 0.1M HCl for 2 h.

### In vitro Drug Release Studies

#### Release studies in buffer I.P. pH 1.2, buffer I.P. pH 7.4 and buffer I.P. pH 6.8

**Fig. 4.**
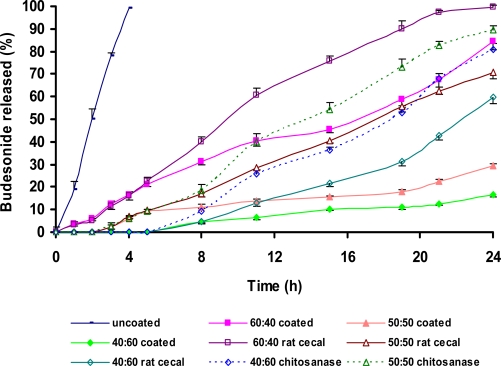
Release of budesonide from tablets containing Eudragit L100-55 alone as binder (uncoated) or after coating with IPC films comprising different ratios of CH:CMTKP in pH progression media, rat cecal contents or in presence of chitosanase

The *in vitro* release of budesonide from the formulated tablets on sequential exposure to pH 1.2 (2 h), pH 7.4 (3 h) and pH 6.8 (19 h) is shown in Fig. 4. The uncoated tablets containing Eudragit L100-55 as binder released 24% budesonide in 2 h (pH 1.2). The remaining drug was released rapidly in pH 7.4 in 4 h. The tablets coated with 60:40 ratio of CH:CMTKP released 6% budesonide in pH 1.2. Additional 21% release was observed upon exposure of tablets to pH 7.4. The IR spectrographs of IPC films (Figs. 3C and D) containing 60:40 ratio of CH:CMTKP had showed a considerable reduction in the intensity of peaks at 1407 cm^−1^ and 1560 cm^−1^ indicating weakening of interaction between −NH_3_^+^ groups of CH and −COO^−^ groups of CMTKP.

The tablets coated with 50:50 or 40:60 ratio of CH:CMTKP did not release budesonide in pH 1.2. However, exposure to pH 7.4 released ∼9% budesonide from tablets coated with 50:50 ratio of CH:CMTKP. It is important to note that tablets coated with 40:60 ratio did not release budesonide at this pH The observation of <10% drug released in acidic media [17] indicated fulfillment of drug release criteria expected of enteric tablets. This was supported by the observation that there was no decrease in the intensity of the peaks characteristic of CH-CMTKP interpolymer complexation in the IR spectrographs of IPC films prepared from 50:50 (Figs. 3E and F) as well as 40:60 (Figs.3G and H) ratios of these polymers after their exposure to pH 1.2 or pH 7.4. Hence, the ability of the 50:50 or 40:60 (CH:CMTKP) IPC film coating in restraining the release of budesonide from tablets can be attributed to the resistance of the complexation between −NH_3_^+^ of CH and −COO^−^ of CMTKP to pH 1.2. It may be highlighted that the use of Eudragit L100-55 as binder could not control the release of budesonide from uncoated tablets. However, coating these tablets with CH-CMTKP (50:50 or 40:60) IPC films restrained the release of budesonide in pH 1.2. Hence, CH-CMTKP IPC films can be suggested to play a pivotal role in modulating the release behavior of the tablets.

**Fig. 5.**
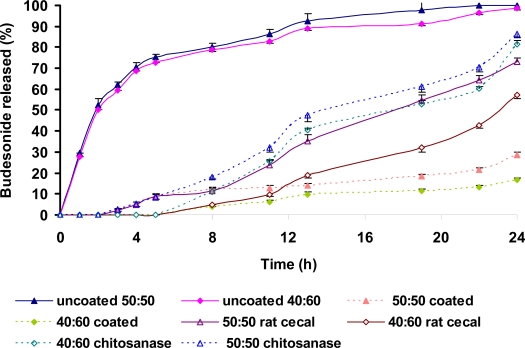
Release of budesonide from tablets containing 50:50 or 40:60 ratio of CH:CMTKP as binder alone (uncoated) or coated with respective IPC film in pH progression media, rat cecal contents or in the presence of chitosanase

The *in vitro* release profiles of budesonide from various batches of tablets containing 50:50 or 40:60 ratio of CH-CMTKP as binder and coated with the respective ratio of their aqueous mixture on sequential exposure to pH 1.2 (2 h), 7.4 (3 h) and 6.8 (19 h) are depicted in Fig. 5. The uncoated tablets containing 50:50 or 40:60 ratio of CH:CMTKP as binder, released, respectively 52% and 50% budesonide in pH 1.2. Further, they released approximately 75% and 72% drug, respectively, in pH 7.4. This, indicated that a combination of CH:CMTKP when used alone as a binder (10% w/w) was not able to restrain the release of budesonide in stomach and small intestine. However, coating these tablets with the IPC films of respective ratio of CH:CMTKP restrained the release of budesonide in conditions mimicking stomach (pH 1.2). Further, the tablets containing 50:50 ratio of CH:CMTKP as binder and coated with the same solution released approximately 9% budesonide in pH 7.4. These tablets released 28% budesonide in pH 6.8 till 24 h. The tablets containing 40:60 ratio of CH:CMTKP as binder and coated with the same solution did not release budesonide in pH 7.4. The total budesonide released in pH 6.8 after 24 h was found to be 16%. The release profiles of budesonide from tablets containing Eudragit L100-55 or CH-CMTKP as a binder and coated with respective CH:CMTKP (50:50 or 40:60) solution were not significantly different.

#### Release studies in the presence of phosphate buffer I.P. pH 6.8 containing rat cecal contents or chitosanase enzyme

The release profiles of budesonide from CH-CMTKP coated tablets containing Eudragit L100-55 or CH-CMTKP solution as binder on sequential exposure to pH 1.2, 7.4 and 6.8 containing rat cecal contents are depicted in Figs. 4 and 5. The release of budesonide in the presence of rat cecal contents in the dissolution medium was significantly (p<0.05) increased as compared to that in its absence. The *in vitro* drug release from tablets containing Eudragit L100-55 as binder and coated with 60:40, 50:50 or 40:60 ratio of CH:CMTKP after 24 h in the presence of rat cecal contents was enhanced to 99%, 70% or 59%, respectively, from 84%, 36% or 25% in the absence of rat cecal contents (Fig. 4). The final exposure of tablets containing 50:50 ratio of CH:CMTKP as binder and coated with the same solution to rat cecal contents released 74% budesonide as compared to 30% in the absence of rat cecal contents. Similarly, the tablets containing 40:60 ratio of CH:CMTKP as binder and coated with the same solution showed an increase in the release of budesonide from 19% to 60% in the presence of rat cecal contents (Fig. 5). The increase in the budesonide release in the presence of rat cecal contents demonstrated the vulnerability of CH:CMTKP IPC to the polysaccharidases present in the colon. Although, the presence of rat cecal contents in the dissolution media enhanced the amount of budesonide released, complete release of budesonide was not observed even after 24 h (Figs. 4 and 5) from tablets coated with CH:CMTKP 50:50 or 40:60. This could be due to the reduction in the activity of polysaccharidases when used over long duration of time during *in vitro* testing. A logical approach for releasing more amount of drug from polysaccharide-based drug delivery systems is to use higher concentrations of rat cecal contents in the dissolution medium. Li-Fang *et al.,* (2009) have reported 60% theophylline release from chitosan/Kollicoat SR 30D coated tablets in the presence of 4%, w/v rat cecal contents [18]. Tavakol *et al.* (2009) used 20%, w/v rat cecal content and observed an increase in the amount of sulfasalazine released from chitosan coated calcium alginate beads. The increase in the amount of sulfasalazine released was found to be less for the tablets coated to higher weight gain [19]. The quantity of fecal matter in humans to which the dosage form might be exposed is manifolds greater than what was used in the present study. Hence, incomplete release of budesonide in the presence of rat cecal contents during these *in vitro* dissolution studies is not unexpected [20].

The presence of chitosanase increased the amount of budesonide released from the coated tablets. The final exposure of tablets coated with 50:50 or 40:60 ratio of CH:CMTKP to pH 6.8 containing chitosanase for 19 h enhanced the amount of budesonide released from tablets containing Eudragit L100-55 as binder to 92.43% and 83.25% as compared to 70.5% and 59.8%, respectively, in the presence of rat cecal contents (Fig. 4). Similarly, the presence of chitosanase in the dissolution media increased the release of budesonide from the tablets coated with IPC film comprising 50:50 or 40:60 ratio of CH:CMTKP containing respective ratio of CH:CMTKP as binder to 89% and 83% as compared to 74% and 57%, respectively, in the presence of rat cecal contents (Fig. 5). The availability of the free amino group in CH is a prerequisite for its hydrolysis by chitosanase enzyme [21]. However, since the amino group of CH molecule is ionically complexed with –COO^−^ groups of CMTKP, complete hydrolysis of CH can not be expected. This seems to be responsible for incomplete (∼90%) budesonide release from IPC film coated tablets.

### Mechanism and kinetics of drug release from coated budesonide tablets

The release kinetics of budesonide from tablets containing Eudragit L100-55 or CH-CMTKP solution as binder and coated with 50:50 or 40:60 ratio of CH:CMTKP was analyzed by Korsmeyer–Peppas model [22]. The value of r^2^ was found to lie above 0.9 in all the cases. The value of release exponent was more than 1.0 in all the cases, indicating that the drug release can be ascribed to a Super Case II transport. Super Case II transport is reported to be exhibited when diffusion and relaxation rates are comparable [23, 24]. The main reason for this observation is erosion of the polymers. However, release of budesonide from these tablets was observed to follow zero order kinetics during sequential exposure of tablets to progressive pH media, rat cecal contents and in presence of chitosanase enzyme (Tab. 2). Hence, it can be suggested that the interpolymer complexation between CH and CMTKP was resistant to different pH media and release of budesonide occurred due to slow erosion of polymers.

### Stability Studies

The tablets coated with 50:50 or 40:60 ratio of CH:CMTKP containing Eudragit L100-55 or CH-CMTKP as binder were found to exhibit complete physical and chemical stability on storage at 40°C/ 75% RH. There was no change in the color and weight of the tablets. The value of *f1* was found to be 2.98 and that of *f2* was found to be 95.63, this indicated that the coated tablets were not affected adversely on storage.

### Pharmacokinetic studies

#### In vivo drug release

**Fig. 6.**
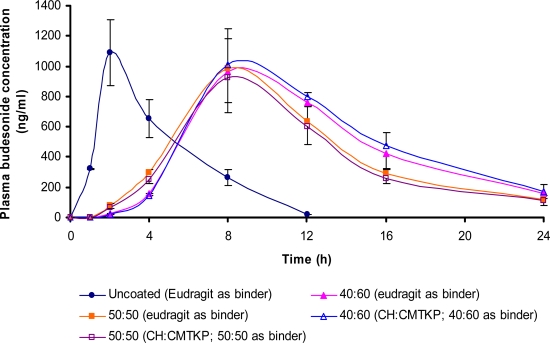
Pharmacokinetic profile of budesonide following oral administration of tablets containing Eudragit L100 55 as binder without coating; coated with aqueous solution containing CH:CMTKP in the ratio of 40:60 or 50:50; containing CH:CMTKP (40:60) as binder and coated with the same solution or containing CH:CMTKP (50:50) as binder and coated with the same solution.

The plasma concentration time profiles of budesonide uncoated tablets as well as of tablets coated with 50:50 or 40:60 ratio of CH:CMTKP containing respective CH-CMTKP solution or Eudragit L100-55 as binder following oral administration to rats is depicted in Fig. 6. The uncoated budesonide tablets containing Eudragit L100-55 as binder released budesonide within 1 h of administration, suggesting that Eudragit L100-55 alone was not able to delay the drug release. The plasma concentration of budesonide was found to rise quickly after administration of uncoated tablets and Cmax of 1091.99 ng/ml was achieved in 2 h. However, after administration of tablets containing Eudragit L100-55 or CH-CMTKP solution as binder and coated with 50:50 or 40:60 ratio of CH:CMTKP, budesonide was detectable in plasma after 2 h of administration. The time to achieve Cmax after oral administration was delayed to 8 h for coated tablets. This strongly indicated that the IPC films were able to inhibit the release of budesonide in gastric pH. However, the plasma budesonide concentration in rats administered with coated tablets (50:50 or 40:60 ratio of CH:CMTKP) rose steadily after 4 h and then declined after 8 h thus suggesting that the polymers were susceptible to degradation by the polysaccharidases present in the colon.

A comparison of rate (Tmax) as well as extent (AUC) of budesonide absorbed from tablets coated with CH-CMTKP IPC films containing Eudragit L 100-55 as binder or those containing CH-CMTKP solution as binder did not reveal any significant difference (p<0.05) (Tab.3). This indicated that CH-CMTKP solution as binder performed in a manner similar to Eudragit L100-55

### Pharmacodynamic Studies

#### Colon/Body weight ratio

**Fig. 7.**
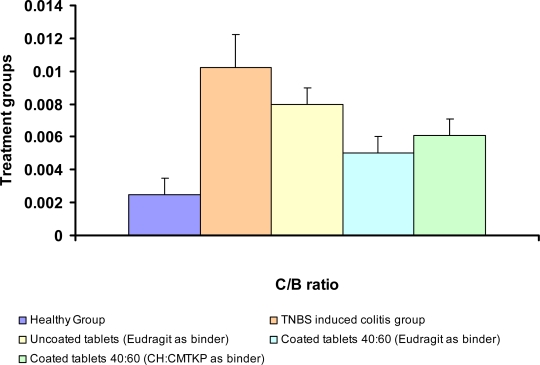
Colon/Body weight ratio determined after 5 days of administration of different formulations. Data presented is mean ± SD (n= 3 animals/ group)

The Colon/Body weight (C/B) ratio in rats was calculated for quantitative evaluation of the inflammatory colitis. The C/B ratio after intracolonic administration of TNBS was significantly more as compared to the group receiving normal saline (p<0.05). The C/B ratio after oral administration of uncoated or IPC film coated budesonide tablets containing Eudragit L 100-55 or CH-CMTKP (40:60) as binder was significantly decreased (p<0.05) as compared to the TNBS-induced colitis groups (Fig. 7). Further, a better therapeutic effect was observed after administration of coated budesonide tablets as compared to uncoated budesonide tablets. This could be attributed to the release of budesonide in colon from the coated tablets. The five day administration of coated tablets (40:60; CH:CMTKP) could not reduce the C/B ratio in the TNBS induced colitic rats to a level comparable to normal rats. Nevertheless, the coated tablets were almost 2-fold more effective than uncoated tablets.

### Histopathological studies

**Fig. 8.**
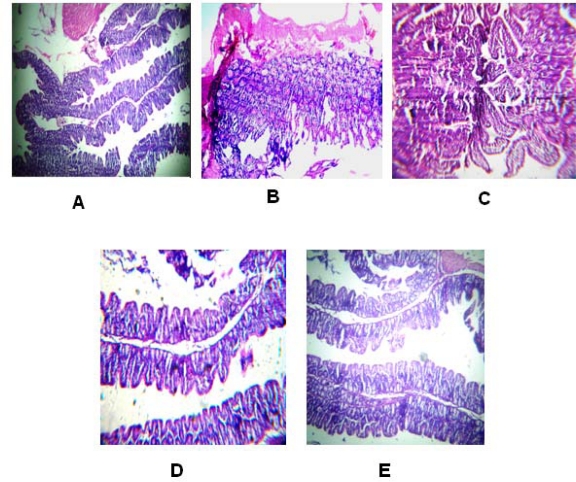
Light microscopic photographs of colon: normal colon (A); TNBS induced ulcerative colon (B); after treatment with : uncoated budesonide tablets containing Eudragit as binder (C); coated budesonide tablets containing Eudragit as binder and coated with 40:60 ratio of CH:CMTKP IPC film (D); coated budesonide tablets containing CH:CMTKP (40:60) as binder and coated with the same IPC film (E)

Fig. 8 shows the histology of normal colon. The four layers of colon wall are mucosa, submucosa, muscularis externa and serosa. The section of the colon shows temporary folds of the mucosa and submucosa (Fig. 8A). As is evident from Fig 8B, the ulcerative colitis induced colon showed shedding of epithelium and lymphocytic infiltration in lamina propria. The inflammation was spread over the mucosa, submucosa, muscle layer and serosa. Oral administration of budesonide tablets containing Eudragit L 100-55 or CH-CMTKP as binder and coated with 40:60 ratio of CH:CMTKP resulted in a marked decrease in the extent and severity of colonic damage. The histopathological features of colon clearly indicated that the morphological disturbances associated with TNBS administration were corrected after five days of oral administration of these tablets (Figs. 8D and E). However, in rats treated with uncoated tablets containing Eudragit L100-55 as binder, lymphocytic infiltration as well as broken and intact epithelial lining was evident (Fig. 8C), suggesting decreased effectiveness of uncoated budesonide tablets in rectifying the ulcerative colitis induced by TNBS administration.

## Conclusion

The results of the present investigation revealed distinct advantage of coating tablets with IPC films containing 50:50 or 40:60 ratio of CH:CMTKP as compared to uncoated tablets granulated with Eudragit L100-55 or CH-CMTKP solution in restraining the *in vitro* release of budesonide in gastric pH. These tablets released budesonide under conditions mimicking large intestine in zero order fashion through 19 h during dissolution studies. Although, the release of budesonide in rats was delayed, the peak plasma concentration was comparable to that obtained after oral administration of uncoated tablets. The ability of the IPC films in providing the observed release characteristics to budesonide core tablets was correlated with the ability of −NH_3_^+^ groups of CH to form complex with −COO^−^ groups of CMTKP and the stability of these complexes in acidic media. The use of enteric polymer as binder offers no advantage for protecting the drug release in stomach. The tablets coated with 40:60 ratio of CH:CMTKP can be envisaged to offer a great promise for colon delivery of budesonide, thereby providing drug concentration in the distal part of gastrointestinal tract for longer durations for effective therapy of IBD.

## Experimental

### Materials

Budesonide was received as a gift sample from Ranbaxy Research Labs, Gurgaon, India. TKP was received as gift sample from Encore Natural Polymers Pvt. Ltd., Ahmedabad, India. CH was purchased from Indian Sea Foods Ltd., Cochin, India. Chitosanase (Product No. C9830, enzyme activity 192.68 units/mg) was purchased from Sigma-Aldrich, Mumbai, India. Ammonium acetate, acetic acid were of analytical grade and were purchased from Qualigens Fine Chemicals, India. Acetonitrile and potassium dihydrogen orthophosphate of HPLC grade were purchased from Merck India, Ltd. All reagents and chemicals were of analytical grade and used as received.

### Procedure for carboxymethylation of TKP and Characterization by IR

Carboxymethylation of TKP was carried out using the method reported by Goyal *et al*. [25]. TKP (0.05 mol) was dispersed in 80 ml alkaline aqueous methanol (0.158 mol sodium hydroxide). To this dispersion monochloroacetic acid (0.09 mol) was added in solid form with continuous stirring for 15 min. The flask was immersed in a thermostatic water bath and the temperature was maintained at 70°C for 60 min. The contents of the flask were shaken occasionally during the course of the study. The reaction product was filtered, dissolved in water and neutralized with dilute acetic acid. The reaction product was precipitated in ethyl alcohol and washed twice with aqueous methanol (80 %, v/v) followed by pure methanol. The products were initially dried at room temperature and then in vacuum oven at 40°C for 4 h to obtain carboxymethyl tamarind kernel powder (CMTKP). The degree of substitution of CMTKP was determined by titrimetric method [12]. The CMTKP was characterized by FTIR analysis.

### Studies on CH-CMTKP Complexes

#### Stoichiometry of the polymer complexes

CH solution was prepared in 1.5% v/v acetic acid. CMTKP solutions were separately prepared by hydrating them in distilled water. Both the solutions were at 25°C to obtain 80:20, 70:30. 60:40, 50:50, 40:50, 30:70 and 20:80 ratio of CH:CMTKP. The samples were incubated at 37°C for 24 h. The samples were then centrifuged at 15000 rpm. The viscosity of the supernatant solution was determined using Brookefield RVDV II Pro Viscometer, UK (Spindle 21).

### Preparation of CH-CMTKP interpolymer complexed films

CH (375 mg) was dissolved in 15 ml solution of 3%, v/v acetic acid. To this mixture 8 ml of 5 M-ammonium acetate was added. CMTKP (375 mg) was separately dissolved in 7ml distilled water and slowly added with stirring to CH solution. This mixture was poured in petriplates and dried at 50 °C for 48 h. Films with a total polymer content of 2.5%, w/v containing 70:30, 60:40, 50:50, 40:60 or 30:70 ratio of CH:CMTKP were prepared using this method. The dried films were stored in a desiccator until use.

### Characterization of IPC films

#### FTIR analysis

CH, CMTKP and IPC films formed by drying admixtures containing different ratios of CH:CMTKP were subjected to FTIR analysis (Perkin Elmer RXI, USA). The fresh films were sequentially exposed to pH 1.2 buffer IP for 2h and pH 7.4 buffer IP for 22 h. The exposed films were dried at 50°C for 24 h and subjected to FTIR analysis.

#### Swelling Index Measurement

The swelling index of the IPC films after exposure to different pH was determined by sequentially immersing the films in pH 1.2 for 2 h and pH 7.4 for 22 h. The swelling index was calculated according to the formula

$$\text{Swelling}\;\text{Index}=\frac{W2-W1}{W1}$$

Where, W1 is the initial weight of the film and W2 is the weight of the swollen film.

### Preparation of core Tablets

Tablets (average weight 25 mg) containing 3 mg of budesonide were prepared by wet granulation technique. Budesonide and Avicel^®^ pH 102 were granulated using Eudragit^®^ L100-55 (alcoholic solution) or CH:CMTKP solution (10% w/w) as binder. The granules were passed through #16 and dried at 50 ± 2°C to 2–3% w/w residual moisture content. The dried granules were passed through #20 sieve and fines were retained on #44 sieve. 10%, w/w of fines was mixed with the granules. Magnesium stearate (1%, w/w) was added to the granules. Tablets were compressed using biconvex punches in a six station rotary tablet compression machine (A K Industries, M207, Nakodar, India). These tablets were tested for dimensions (axial and radial diameters), hardness, friability and weight variation.

### Testing of uncoated tablets

The axial and radial diameters of ten compressed tablets of each batch were determined by using electronic digital vernier calipers. Hardness of ten tablets was determined with the help of Pfizer hardness tester (Campbell Electronics, Mumbai). The friability test, weight variation test and disintegration test were performed in accordance with the method prescribed in USP 30, NF 25 [17].

### Coating of budesonide tablets

The formulated budesonide tablets containing Eudragit L100-55 or CH:CMTKP solution as binder were coated with aqueous solutions containing different CH:CMTKP ratios (Composition same as IPC film) to obtain a weight gain of 10%, w/w. The total polymer concentration was kept constant at 2.5%, w/v. A separate batch of compressed tablets was coated with 2.5%, w/v solution of CH. The coating solution was sprayed at a rate of 5 ml/min with the help of peristaltic pump using a spray gun of 1 mm nozzle (Electrolab, PP201V, Mumbai, India) in a coating pan (12″diameter) being rotated at 18 rpm (AK Industries, M1107, Nakodar, India). Compressed air was introduced at a pressure of 1.5 kg/cm^2^. The inlet air temperature was maintained at 60°C. The inner surface of coating pan was modified by attaching inert tubes (8 mm diameter) from the centre to the periphery for easy rolling of tablets thereby ensuring efficient mass transfer of polymer.

The coated tablets were also evaluated for weight variation, disintegration time. Further, the axial and radial diameters were measured as described above.

### In vitro release kinetics of budesonide from coated tablets

*In vitro* release of budesonide from coated tablets was evaluated out using USP 30-NF25 (Dissolution Apparatus 1-basket method) utilizing temperature of 37±0.5°C with constant stirring rate of 50 rev/min in a pH progression media containing 0.5% v/v Tween 20 [26] to maintain sink conditions. pH progression consisted of exposure of tablets to buffer pH 1.2 IP for 2 h followed by buffer pH 7.4 IP for 3 h and buffer pH 6.8 for further period of 19 h. Dissolution studies were also carried out in pH 1.2 (2 h), pH 7.4 (3 h) followed by pH 6.8 (19 h) containing rat cecal contents (4%, w/v) or chitosanase to check the vulnerability of the CH-CMTKP IPC films to polysaccharidases produced by the colonic bacteria. The chitosanase (192.68 units/mg) was dissolved in sodium acetate (50 mM) buffer pH 5.5 to yield a solution containing 0.05 units of chitosanase in 100 ml of dissolution media. These solutions were prepared immediately before use. Aliquots (5 ml) from the dissolution media were withdrawn at predetermined intervals and immediately analyzed for budesonide using HPLC. An equal volume of respective buffer containing Tween 20 (0.5%, v/v) was replaced after each sampling.

### HPLC analysis

The samples obtained from dissolution studies were filtered through 0.45 μ nitrocellulose filters (Millipore, USA) and manually injected (20 μL) using Rheodyne injector for HPLC analysis. The stationary phase consisted of C8 column (150 mm×4.6 mm, 5 μ). Acetonitrile and 25 mM phosphate buffer (pH 3.2) in the ratio of 60:40 v/v at a flow rate of 1 ml/min served as mobile phase [27]. Data acquisition and processing was performed by using Empower 2 software (Waters, Austria).

### Mechanism and Mathematical Modeling for Drug Release

The mechanism of drug release during dissolution studies in pH progression media, in the presence of rat cecal contents or in the presence of chitosanase was evaluated by using the Korsmeyer equation

$$\frac{Mt}{M_{\infty }}=Kt^{n}$$

Where,

Mt/M_∞_ = fractional release of drug,
t = release time,
k = kinetic constant, which incorporates structural and geometric characteristics of the device
n = release exponent, which indicates the kinetic release.

When the exponent *n* takes a limiting value of 0.45, it is the case of diffusion-controlled drug release (Fickian release). Case II transport or relaxation controlled delivery; the exponent *n* is 0.89 for release from cylinders. Values of *n* between 0.45 and 0.89 can be regarded as an indicator for the non-Fickian release or anomalous transport. The non-Fickian kinetics is regarded as couple diffusion/polymer relaxation. In addition, Super Case II kinetics is followed when the values of *n* are greater than 0.89 [22].

### Stability studies

The tablets coated with 50:50 or 40:60 ratio of CH:CMTKP were sealed in glass vials and stored under 45°C/ 75% RH for 6 month. Tablets were taken out after every 15 days and evaluated for weight variation, disintegration time, and *in vitro* drug release. The dissolution data of stored tablets was compared with that of freshly prepared tablets by *f_1_* (*dissimilarity*) and *f_2_* (*similarity*) *analysis*.

### Pharmacokinetic studies

Spargue-dawley rats of either sex weighing 200–300 g maintained on normal diet were used for this study. Rats were divided into seven groups. Each group comprised of four rats for generation of data in one study to ascertain that not more than two blood samples were withdrawn from each rat. Each study was conducted in triplicate. Rats of Group I, Group II and Group III received oral administration of, respectively, uncoated tablets or tablets coated with admixtures containing 40:60 or 50:50 ratio of CH:CMTKP containing Eudragit L100-55 as binder. Rats of Group IV and V received uncoated tablets and tablets coated with IPC films comprising of 50:50 ratio of CH:CMTKP containing the same solution as binder. Similarly, rats of Group VI and VII received uncoated and tablets coated with admixtures containing 40:60 ratio of CH:CMTKP containing the same ratio of CH:CMTKP solution as binder. Blood samples (1 ml) were collected from retro orbital vein at 0, 1, 4, 8, 12, 16 or 24 h, mixed with 40 μl of heparin and subjected to centrifugation at 4000 rpm for 10 min. Upper layer was removed carefully and to 200 μl of plasma, methanol 20 μl and 15 μl of internal standard solution (0.005% w/v clotrimazole in methanol) was added. Ethyl acetate (2 ml) was added and the microcentrifugation tubes were vortexed for 10 min. The tubes were then centrifuged at 4000 rpm for 10 min. The upper layer was aspirated, evaporated and reconstituted with 1mL of mobile phase. 20 μl of this solution was injected for HPLC analysis. The protocol for this study was approved by the IAEC of Punjabi University, Patiala, India.

### Pharmacodynamic evaluation

Sprague Dawley rats (200–250g) of 8–12 weeks age were used in the study. They were fed with standard laboratory chow diet and given water *ad libitum*. The experimental protocol was approved by Institutional Animal Ethics Committee of the Punjabi University, Patiala and the care and handling of the animals were in accordance with the National Institutes of Health guidelines.

#### Induction of colonic inflammation

The rats were fasted for 48 h before the induction of ulcerative colitis. 2,4,6 Trinitro benzene sulphonic acid (TNBS) (20mg) was dissolved in 0.25 ml of 50% v/v ethanol. This solution was instilled to each rat into the colonic part (7 cm from the anus) with the help of a catheter in order to induce ulcerative colitis [28]. The rats were monitored for three days without treatment to allow for the development of ulcerative colitis.

#### Treatment of ulcerative colitis

The rats that weighed 80–100% of their initial weight (before TNBS instillation) were selected after 3 days as the ulcerative colitis positive animals. These rats were divided into four groups. Rats of Group I, Group II received oral administration of, respectively, normal saline, uncoated tablets containing Eudragit L100-55 as binder. Group III and Group IV received oral administration of tablets coated with IPC films comprising 40:60 ratio of CH:CMTKP containing Eudragit L100-55 or respective ratio of CH:CMTKP solution as binder.

All the groups received oral treatment once daily for five consecutive days. The colitis control group received 0.5 ml of normal saline instead of budesonide tablets.

#### Assessment of colonic inflammation

Colonic inflammation following rectal administration of TNBS as well as after different treatments (as detailed above) was assessed by the following parameters.

#### Colon/ body weight ratio

The rats were sacrificed on the sixth day (after five days of tablet administration) and distal colon segments (6 cm length) were resected, opened longitudinally and rinsed with iced phosphate buffer. The ratio of wet weight of the colon specimen to the body weight was calculated for each rat.

#### Histopathological studies

Tissue segments (1 cm in length) were fixed in 10% buffered formalin for histopathological studies. Histopathological studies were carried out using haematoxylin and eosin stains at Department of Pathology, Government Rajindera Hospital, Patiala, Punjab.

## Figures and Tables

**Tab. 1.** Swelling indices of freshly prepared IPC films

**Film Composition (CH:CMTKP)**	**Swelling Index^a^**	
	**pH 1.2**	**pH 7.4**
70:30	2.56 ± 0.11	1.41 ± 0.07
60:40	2.13 ± 0.10	1.16 ± 0.09
50:50	1.57 ± 0.08	1.01 ± 0.05
40:60	1.24 ± 0.05	0.89 ± 0.07
30:70	2.18 ± 0.10	1.24 ± 0.04

a Mean ± S.D of three experiments

**Tab. 2.** Release kinetics of budesonide from tablets containing Eudragit L100-55 or CH:CMTKP as binder and coated with respective CH-CMTKP IPC films

**Binder**	**Coat**	DM*	**Korsemeyer and Peppas equation**	
			**r^2^**	**Order of Release**
Eudragit L100 55	CH:CMTKP 50:50	A	0.9451	Super Case II
		B	0.9534	Super Case II
		C	0.9743	Super Case II

	CH:CMTKP 40:60	A	0.9763	Super Case II
		B	0.9528	Super Case II
		C	0.9778	Super Case II

CH:CMTKP 50:50	CH:CMTKP 50:50	A	0.9127	Super Case II
		B	0.9690	Super Case II
		C	0.9760	Super Case II

CH:CMTKP 40:60	CH:CMTKP 40:60	A	0.9760	Super Case II
		B	0.9675	Super Case II
		C	0.9742	Super Case II

* Dissolution Media; A: pH progression media; B: In rat cecal contents; C: In presence of chitosanase

**Tab. 3.** Pharmacokinetic parameters of budesonide tablets containing Eudragit L100-55 as binder and coated with CH-CMTKP IPC films

**Binder**	**Coat**	**Pharmacokinetic parameters**		
		**T_max_ (h)**	**C_max_ (ng/ml)**	**AUC**

Eudragit L100 55	Uncoated	2	1091.9 ± 218.38	5019
	CH:CMTK P 50:50	8	998.21 ± 229.98	8882
	CH:CMTK P 40:60	8	1009.63 ± 203.6	11531

CH:CMTKP 50:50	CH:CMTK P 50:50	8	1012.65 ± 263.36	9355

CH:CMTKP 40:60	CH:CMTK P 40:60	8	1045.54 ± 203.67	11816
